# Clinical characteristics of 14 pediatric mycoplasma pneumoniae pneumonia associated thrombosis: a retrospective study

**DOI:** 10.1186/s12872-022-03030-9

**Published:** 2023-01-04

**Authors:** Y. Fu, T. Q. Zhang, C. J. Dong, Y. S. Xu, H. Q. Dong, J. Ning

**Affiliations:** Department of Respiratory Medicine, Tianjin Children’s Hospital (Children’s Hospital of Tianjin University), Tianjin, China

**Keywords:** Mycoplasma pneumoniae, Thrombosis, Children

## Abstract

**Objective:**

This study aimed to investigate the clinical characteristics and long-term prognosis of mycoplasma pneumoniae pneumonia (MPP)-associated thrombosis and to gain a better understanding of the diagnosis and treatment of the disease.

**Methods:**

The medical records of 14 children with MPP-associated thrombosis between January 2016 and April 2020 were retrospectively reviewed at the Tianjin Children’s Hospital.

**Results:**

The ages of the patients ranged from 3 to 12 years old. Among the 14 cases, there were five cases of pulmonary embolism, two cases of cerebral infarction, one case of splenic infarction, one case of cardiac embolism, two cases of cardiac embolism with comorbid pulmonary embolism, one case of internal carotid artery and pulmonary embolism, one case of combined internal carotid artery and the cerebral infarction, and one case combined cardiac embolism and lower limb artery embolism. All cases had elevated D-dimer levels. After thrombolysis and anticoagulation therapy, three cases with cerebral embolism still suffered from neurological sequelae. In contrast, the remaining cases did not develop complications.

**Conclusion:**

MPP-associated thrombosis can occur in any vessel of the body. Thrombosis-associated symptoms may be complex and non-specific. Elevated D-dimer levels in a child with refractory mycoplasma pneumoniae pneumonia should raise suspicion of thrombosis. The long-term prognosis of thrombosis was favorable after the timely administration of anticoagulant therapy.

## Introduction

Mycoplasma pneumoniae is the most common pathogen in the lower respiratory tract infection of children, accounting for 10–40% of all hospitalized children [1] and 2–12% of adults [2–4] with community-acquired pneumonia. As is well documented, roughly 25% of children with MPP manifest other systemic symptoms [5]; cardiac manifestations are among the most frequently reported extrapulmonary manifestations, including pericarditis, endocarditis, and myocarditis [6–9]. Moreover, Stevens–Johnson syndrome and erythema multiforme have frequently been reported in association with mycoplasma pneumoniae infection [10], while hepatitis is relatively common [11]; Despite pancreatitis being reported only sporadically [12, 13], it is often associated with generalized severe conditions. Notably, autoimmune hemolytic anemia (cold agglutinin disease) is the most prevailing indirect-type extrapulmonary manifestation due to mycoplasma pneumoniae infection [5, 14]. Meanwhile, hemophagocytic syndrome is generally recognized as a cytokine-storm disorder and has been rarely reported in association with mycoplasma pneumoniae infection [15, 16]. Previous studies have also established that rhabdomyolysis may be associated with MPP [17]. Otitis media, which occurs predominantly in younger children, is also an extrapulmonary manifestation of mycoplasma pneumoniae infection [5, 14]. Lastly, few cases of glomerulonephritis [18, 19] and IgA nephropathy [20] have been documented in association with mycoplasma pneumoniae infection.

Thrombosis is one of the extra-respiratory manifestations associated with Mycoplasma pneumoniae infection. MPP complicated by thrombosis is uncommon [21–28].Embolism is prone to severe sequelae and even lifethreatening if not timely diagnosed and treated. However, there have been only a few reported cases of MPP-associated thrombosis, and data on clinical characteristics are scarce. With the increase of awareness of this disease, we found thrombosis in MPP was not rare. Mycoplasma pneumoniae infection and thrombosis may occur in different parts of the body, and its clinical manifestations are complex and diverse, sometimes influencing the prognosis of the disease. This retrospective study aimed to describe cases of MPP complicated with thrombosis in an effort to advance knowledge of the diagnosis and treatment of this disease.

## Methods

### Study population

**Fig. 1:**
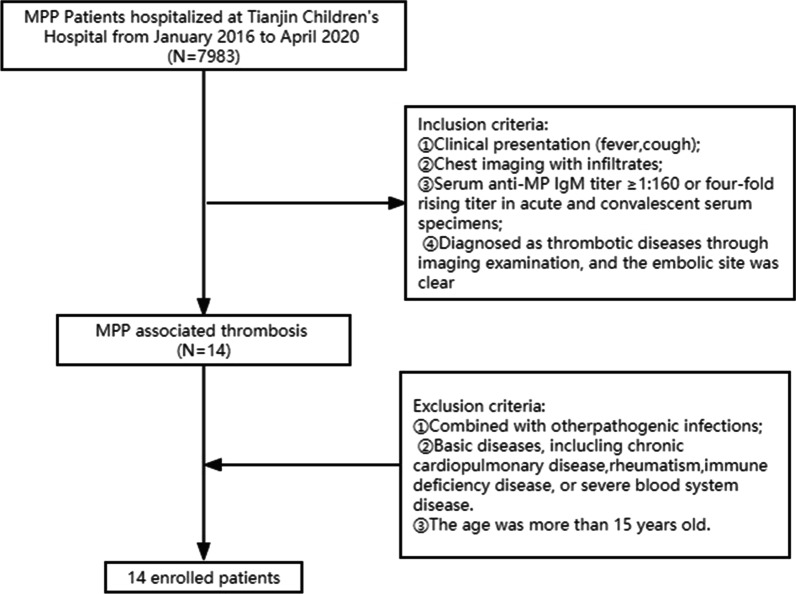
Patient enrollment flow chart

This retrospective analysis included 14 patients with mycoplasma pneumonia infection complicated with thrombosis who were treated in Tianjin Children's Hospital between January 2016 and April 2020. A detailed summary of subject enrollment is presented in Fig. 1. The study was approved by the Ethics Committee of Tianjin Children’s Hospital. The medical records of all subjects were anonymously reviewed. Clinical characteristics, including clinical presentation, the levels of inflammatory markers such as C-reactive protein (CRP) and lactate dehydrogenase (LDH), and the results of blood coagulation tests were collected. In addition, the findings of bronchoscopy, contrast-enhanced lung CT, echocardiography, blood vessel ultrasonography, and treatment outcomes, were also retrospectively analyzed.

### Diagnostic criteria

The diagnostic criteria of MPP were as follows [29]: (1) clinical presentation (fever and cough); (2) chest imaging with infiltrates; (3) serum anti-MP IgM titer ≥ 1:160 or four-fold rising titer in acute and convalescent serum specimens. In addition, the diagnosis of thrombotic diseases was confirmed through imaging examination. The exclusion criteria were as follows: (1) Disease complicated with other pathogenic infections. (2) Other severe diseases, including chronic cardiopulmonary diseases, rheumatism, autoimmune diseases, or severe hematological diseases. (3) Patients older than 15 years old.

### Statistical analyses

SPSS-Version 24 (IBM Corp., Armonk, NY, US) was utilized for statistical analysis of the collected data. All values presented were expressed as mean ± standard deviation (SD).

## Result

### Baseline data

As presented in Table 1, 14 patients, including 5 males and 9 females, with ages ranging from 3 to 12 years and a mean age of 7.5 years, were finally enrolled. All patients were otherwise physically healthy.

**Table 1:** Results of auxiliary examinations of the patient

Patient No	Gender	Age (years)	Duration of diagnosis (days)	Affected vessels	D-Dimer (mg/L)	CRP (mg/L)	N%	LDH (U/L)	MP IgM titer
1	Female	9	22	Right lower pulmonary artery	20	146	82	1087	1:640
2	Female	7	13	Left upper pulmonary artery, right lower pulmonary artery	11.8	179.7	89	1052	1:320
3	Male	7	14	Left middle cerebral artery	10.4	170	87	720	1:640
4	Female	9	6	Left anterior cerebral artery	6.2	50	85	911	1:160
5	Female	12	9	Right ventricle, right deep femoral artery, right posterior tibial artery, right dorsum pedis	6.8	59	78	631	1:160
6	Male	3	5	Right internal carotid artery, right middle cerebral artery, right anterior cerebral artery	2.2	90	82	874	1:640
7	Female	5	16	Bilateral inferior pulmonary arteries	7.2	86	80	719	1:160
8	Female	8	8	Right ventricular thrombosis, left lower pulmonary artery	13.9	164	92	851	1:160
9	Female	9	16	Right ventricular thrombosis	4.56	121	89	577	1:160
10	Male	3	17	Left internal jugular vein, left pulmonary artery trunk and left superior and inferior pulmonary artery	6.3	93.2	73	389	1:1280
11	Female	12	6	splenic artery	3.6	53.6	82	563	1:1280
12	Female	5	9	Right inferior pulmonary artery	5	122.5	84.5	963	1:640
13	Male	7	20	Branches of right atrium and right inferior pulmonary artery	0.5	54.6	83	545	1:640
14	Male	8	14	Bilateral inferior pulmonary arteries	0.56	200	93	857	1:640

*CRP* C-reactive protein, *N* neutrophil, *LDH* lactate dehydrogenase, Normal range: CRP < 8 mg/L; LDH < 300 IU/L; ALT < 40 IU/L; D-dimer < 0.5 mg/L

### Clinical characteristics of patients

All children presented with a persistently high fever and cough (n = 14, 100%). The disease duration prior to hospitalization in our department was 5–18 days. Thrombosis occurred between the 5th and the 22nd day after the disease onset. The nature of the embolism in the 14 children was: pulmonary in 5 cases, cerebral in 2 cases, splenic in 1 case, right ventricular in 1 case, pulmonary and cardiac in 2 cases, pulmonary and internal jugular vein in 1 case, internal carotid artery and cerebral in 1 case, and lower extremity artery and cardiac thrombosis in 1 case. In addition to the primary disease manifestations, secondary signs of pulmonary embolism in children comprised cough, dark red blood clots, bloody sputum, chest pain, transient dyspnea, neck pain, and so on. Contrastingly, the primary manifestations of children with cerebral embolism included rapid loss of consciousness, limb paralysis and weakness, slurred speech, incomprehensible pronunciation, involuntary movement, and partial eyelid closure, among others. At the same time, children with splenic embolism exhibited severe pain in the umbilical and left hypochondriac areas, as well as vomiting. Patients with arterial embolism of lower limbs developed cyanosis of foot skin, small ecchymoses, toe pain, low peripheral temperature, a feeble pulse of the arteria dorsalis pedis, and rigid movement. Children with cardiac thrombosis mainly suffered from chest pain. Children with internal jugular vein embolism showed an unequal height of shoulders. Interestingly, the 2 cases with pulmonary embolism were asymptomatic.

#### Laboratory data

The mean peripheral white blood cell (WBC) counts of the 14 children was 12.5 ± 3.4 × 10^9^/L, while the mean neutrophil (N) percentage was 0.84% ± 0.05%. The mean platelet counts were 399.2 ± 93.0 × 10^9^/L, and the average C-reactive protein (CRP) level was 113.5 ± 49.7 mg/L. Meanwhile, the mean lactate dehydrogenase (LDH) level was 767.1 ± 200.4 IU/L and was as high as 1087 U/L in 1 case. Elevated alanine aminotransferase (ALT) levels were detected in 5 patients. Finally, the levels of ferritin (FER), D-dimer, and fibrinogen were 519.2 ± 197.9 g/L, 7.6 ± 5.1 mg/L, and 5.0 ± 1.7 m/L, respectively.

Five cases were examined for bronchoalveolar lavage fluid mycoplasma PCR resistance mutation sites 2063/2064, and all tested positive. Six of nine children tested positive for anti-nuclear antibodies (ANAs), and all returned to within the normal range after 3 months. Antiphospholipid antibody tests (anticardiolipin antibody, lupus anticoagulant, Anti-beta-2-glycoprotein) was performed on 3 children. One case tested positive for lupus anticoagulant, one case was positive for anticardiolipin antibody IgA, and the remaining cases were negative; the level for one case returned to normal after 3 months, whereas the other patient was lost to follow-up.

#### Radiological examination

Pulmonary CT scan displayed pulmonary consolidation in all cases; pulmonary CT showed that the bronchi were not unobstructed in 7 cases. However, atelectasis was noted in 11 cases, and mild to moderate pleural effusion was observed in 13 cases. Additionally, CT demonstrated consolidation and necrosis in 4 cases.

As listed in Table 1, in the 7 children with pulmonary embolism, the main thromboembolic vessels were bilateral lower lobe pulmonary artery and left upper lobe pulmonary artery, of which 2 cases were complicated with right cardiac thrombosis, 1 case was complicated with right ventricular thrombosis, 1 case also suffered from right atrial thrombosis, and 1 case developed left internal jugular vein filling defect. In 3 children with cerebral embolism, the primary embolic vessels were the bilateral middle cerebral arteries and bilateral anterior cerebral arteries, including 1 case complicated with internal carotid artery embolism. There was also one case of right ventricular thrombosis complicated with right external iliac artery embolism.

**Fig. 2:**
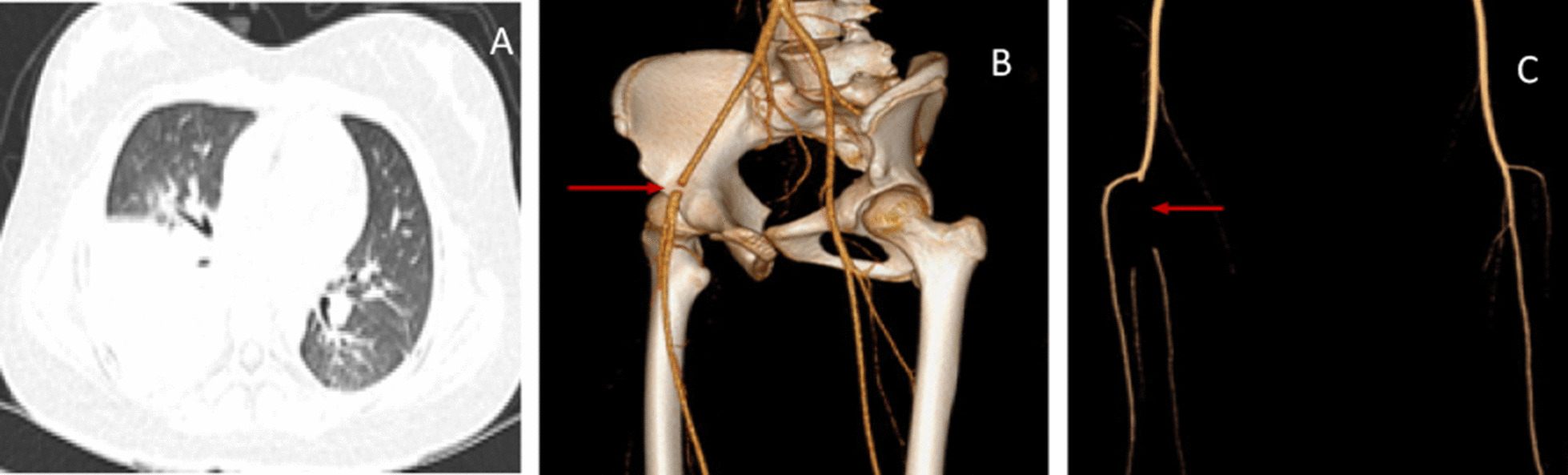
**A** Chest imaging revealed consolidation with high density in the right Lower lung. **B** Lower extremity CTA showed stenosis in of the right external iliac artery. **C** Lower extremity CTA showed that the right posterior tibial artery was not locally visualized.tibial artery

**Fig. 3:**
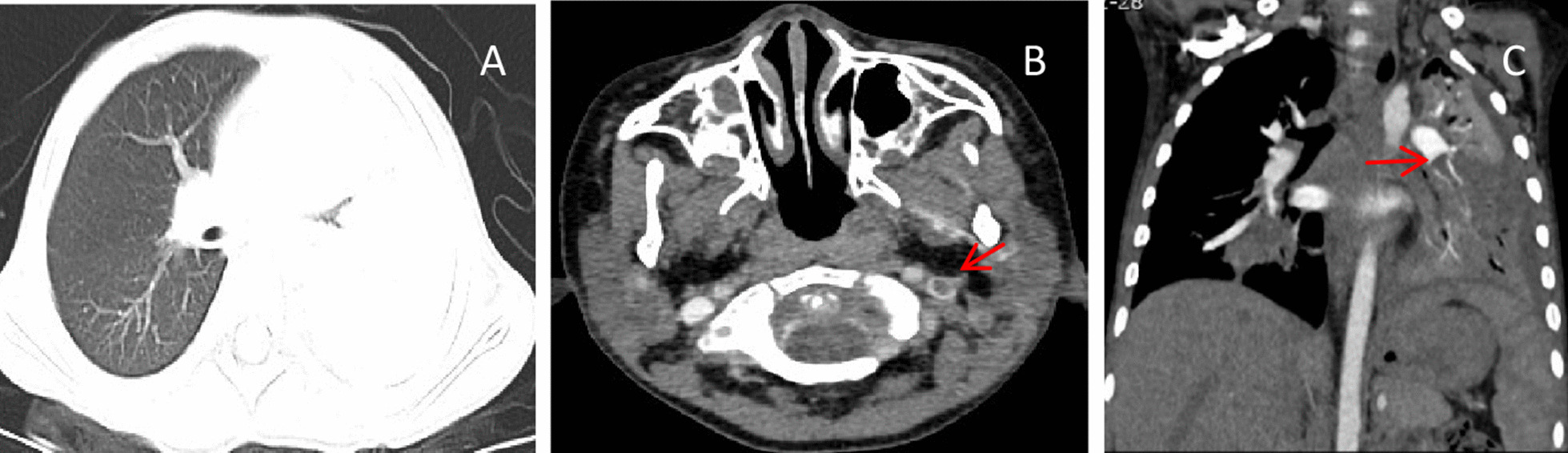
**A** Chest imaging revealed consolidation with high density in the left lung. **B** Neck CTA showed a filling defect in the left internal jugular vein. **C** Chest CTA showed a filling defect in the left pulmonary artery

**Fig. 4:**
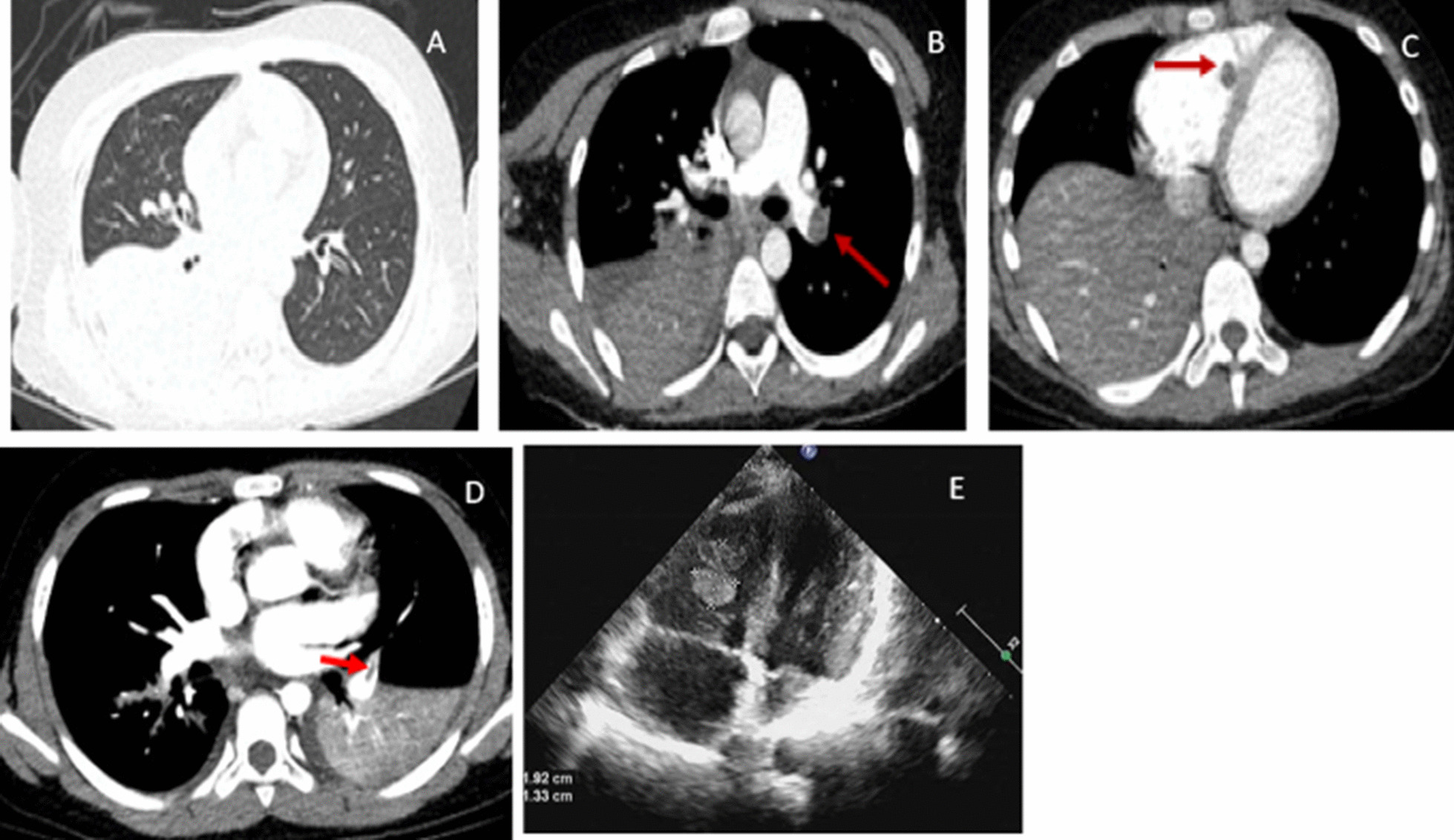
**A** Chest imaging revealed consolidation with high density in the right lower lobe. **B**, **C** Chest CTA showed filling defects in the left pulmonary artery and the right ventricle. **D** Coronal reconstruction of CTA shows filling defect of left pulmonary artery and right pleural effusion (arrow). **E** Echocardiography showed mass-like echo in the right ventricle, about 9 × 6 mm, 13 × 8 mm in size

The radiological findings of embolism varied in different parts of the body. For instance, pulmonary CT angiography (CTA) showed a filling defect in the pulmonary artery and its branches. Contrastingly, brain MRI revealed ischemic foci (patchy aberrant signals) in the frontotemporal basal ganglia. In one case of splenic embolism, CTA illustrated a filling defect in the splenic artery from the beginning of the splenic artery to the splenic hilum. In the case of lower extremity arterial embolism (case 5, Fig. 2), vascular B-ultrasound demonstrated that the arterial blood flow velocity was reduced,the lower extremity CTA showed stenosis in of the right external iliac artery,that the right posterior tibial artery was not locally visualized tibial artery.Echocardiography of cardiac thrombosis (case 9, Fig. 3) exposed multiple right ventricular shadows, swing with heartbeat; CTA showed a nodular filling defect in the right ventricle. CTA showed a filling defect of the left internal jugular vein in a child with internal jugular vein embolism (case 10, Fig. 4). All children had no vascular malformation, as confirmed by imaging examination.

### Bronchoscopy

Seven patients underwent bronchoscopy, which revealed viscous secretion in all patients, mucus plugs in 5 patients, and fibrinous bronchitis in 4 patients.

#### Treatment

All 14 patients were treated with azithromycin, methylprednisolone (2–10 mg/kg/d), and low molecular weight heparin anticoagulation and aspirin to inhibit platelet aggregation. 3 patients were treated with gamma globulin. Moreover, 2 patients with pulmonary embolism complicated with cardiac thrombosis were treated with urokinase. 2 patients with cerebral infarction were treated with a dehydrating agent to lower the intracranial pressure, and those with hepatic injury were treated with hepatoprotective drugs. Lastly, 3 patients with massive pleural effusion underwent chest thoracentesis.

#### Outcomes

No fatalities occurred in this study, and 11 cases with thrombosis-associated symptoms resolved while the laboratory indexes returned within the normal range. In 3 cases of pulmonary embolism, pulmonary CTA revealed no filling defect after 1–3 months, and in 2 cases with cardiac thrombosis, no thrombus was visualized after 0.5–3 months. Three children with cerebral embolism were followed up for 12 months. One of them had enhanced limb activity but was nonetheless affected by sequelae such as tremors and poor motor activities. The remaining two children were regularly treated with rehabilitation exercises in our hospital following discharge. At present, existing symptoms remain, such as unclear pronunciation, unsteady gait, reduced muscle volume, decreased muscular power, postural tremors, and so on. Brain MRA showed the collateral circulation was still not developed, and there had been no improvements since the previous MRA. Cerebral infarction was accompanied by the formation of softening foci.

## Discussion

Pediatric patients with MPP complicated with thrombosis have rarely been described. The incidence of thrombosis in all MMP case was 0.17% in Tianjin Children’s Hospital between January 2016 and April 2020. Cases with mild pulmonary embolism (PE) may be asymptomatic, whereas severe cases may suffer from complex and diverse manifestations, which are challenging to diagnose and generally have a poor prognosis. The diagnosis of RMPP was made based on the fact that children with M. pneumoniae pneumonia after 7 days of treatment with macrolide antibiotics and have aggravated clinical signs, continued fever and aggravated pulmonary imaging findings. All patients met the diagnosis of RMPP.This observation implies that RMPP is the strongest risk factor for MPP-associated thrombosis. Attention should be paid to the manifestations of embolism in these children so as to achieve early diagnosis, active treatment, and an improved prognosis.

The mechanism underlying thrombosis owing to MPP remains elusive. Narita M recommended that the pathological mechanism of extrapulmonary manifestations due to MP infection be classified into three categories; the first is the direct type, in which bacterial cell membrane lipoproteins locally induce the generation of inflammatory cytokines; the second is the indirect type, which involves autoimmunity through the cross-reaction between MP bacterial components and human cells, and the third is the vascular occlusion type, in which the bacterium induces vasculitis and/or thrombosis with or without a systemic hypercoagulable state [30]. Mycoplasma pneumonia may directly cause local thrombotic occlusion by affecting the vascular wall [30]. An autopsy of a patient with acute myocardial infarction revealed that mycoplasma pneumoniae was present in the unstable segments of the intima [31]. In addition, systemic hypercoagulability through the activation of chemical mediators, including complement, may result in thrombotic vessel occlusion [30]. The pathogenesis of M. pneumoniae infection with thrombosis may be correlated with immune damage mediated by infections [32]. Considering that the membrane proteins and glycolipids of mycoplasma pneumoniae share common antigens in the heart, liver, lung, brain, kidney, and smooth muscle tissues of the human body, upon infection of the host with mycoplasma pneumoniae, the corresponding antibodies are produced, and the immune complex activates complement, which attracts neutrophils. Chemokines, which attract white blood cells to invade the lesion, release several inflammatory mediators and lysosomal enzymes, resulting in inflammatory damage in the target organs. Previous studies and case reports have established that patients with thrombosis secondary to mycoplasma pneumoniae infection were positive for anticardiolipin antibodies, β2-glycoprotein antibodies, or lupus anticoagulant antibodies [28, 33]. Anticardiolipin antibody, β2-glycoprotein antibody, and lupus anticoagulant antibody are all antiphospholipid antibodies, which react to phospholipids, phospholipid-protein complexes, and phospholipid-binding proteins [34, 35]. The antiphospholipid antibodies contribute toward the formation of a thrombus [35]. These aforementioned antibodies were transient and became negative in certain patients 3–6 months after initial disease onset [28, 33].

Thrombosis can occur in the vessels of any part of the body (see Table 1). All 14 patients developed thrombosis on days 5–22 and were hospitalized on days 5–18 from the onset. Cerebral and splenic artery embolism were diagnosed earlier, that is, on the 5th to 6th day of the course of the disease. It is possible that these forms of thromboses are often very symptomatic; hence they could be diagnosed earlier than the other forms. The chief manifestations of pulmonary embolism are chest pain, hemoptysis, neck pain, dyspnea, etc. Children with cerebral embolism typically initially suffer from abnormal limb movement and paralysis. The manifestations of lower limb arterial embolism are weak arterial pulsation, reduced skin temperature, cyanosis, inflexible activity, etc. Owing to its insidious onset in some patients, attention should be paid to fluctuations in children's conditions. For those with acute chest pain, hemoptysis, and sputum and whose conditions have not recovered after aggressive treatment, we should be aware of the possibility of thrombosis and promptly consider radiologic examinations.

Herein, 14 children developed complications other than thrombosis. Among them, 13 children developed mild to moderate pleural effusion, 6 developed hepatic dysfunction, and 3 developed rashes. It is worth pointing out that 2 of them had no evident manifestations of embolism during the early and later stages of the disease. Pulmonary imaging revealed necrosis and consolidation of the lobar lung, accompanied by multiple thin-walled or non-walled cavities. Embolism was occasionally discovered via enhanced pulmonary CT. At present, the causes and mechanisms underlying necrotizing pneumonia are principally believed to be related to pulmonary capillaries in the necrotic area being blocked by thrombi, thereby resulting in pulmonary parenchymal ischemia and necrosis under the action of inflammatory factors [36]; the pulmonary parenchyma is ischemic, infarcted, and necrotic, and a cavity is formed after the necrotic matter is discharged [37, 38]. Interestingly, the pulmonary embolus in this study was not consistent with the location of necrotizing pneumonia. We hypothesize that some children may have multiple vascular thrombotic occlusions except for large vessel embolism.

The peak value of venous blood CRP, LDH, FER, and the percentage of neutrophils in the 14 children were significantly increased. We postulate that this may be related to the serious systemic inflammatory response triggered by mycoplasma pneumoniae infection. D-dimer levels were elevated as well, suggesting a hypercoagulable state [39]. One patient had a marginal increase in D-dimer level (0.5 mg/L), potentially attributable to a lack of timely monitoring. The eighth child had a D-dimer level of 0.5 mg /L on admission. On the second day of admission, he suffered from neck pain and shortness of breath, and the D-dimer level increased to 11 mg/L. Cardiac ultrasound and pulmonary CTA were performed to diagnose cardiac and pulmonary artery thrombosis. Therefore, D-dimer levels should be dynamically monitored in children at high risk of thrombosis to guide diagnosis and treatment. D-dimer is the smallest fragment of fibrin degradation products. An increase in its level signifies a hypercoagulable state and active fibrinolysis, which is crucial for the early diagnosis of thrombotic diseases. Indeed, D-dimer has a high negative predictive value in the diagnosis of thrombotic diseases. D-dimer < 0.5 mg/L as the cutoff value in conjunction with clinical manifestations is an important auxiliary test for screening patients with suspected pulmonary embolism [40]. For pulmonary embolism, the diagnostic sensitivity of plasma D-dimer can attain 92–100% [41]. Beijing Children's Hospital observed that the mean D-dimer level of 43 children with MPP-associated thrombosis was 11.1 ± 12.4 mg/L [28].

Angiography is the gold standard for the diagnosis of deep venous thrombosis and pulmonary embolism; however, its clinical applicability in children is limited, attributable to its invasive nature and potential risks [42].CTA is a non-invasive method that can be applied to the examination of systemic blood vessels, and its tendency to replace DSA as the gold standard is becoming increasingly apparent. For children with cerebral infarction, MRI + MR angiography (MRA) can clearly delineate the abnormal signal and the development of vascular stenosis and occlusion so as to guide clinical diagnosis and treatment. MRI is the preferred choice for children with cerebral infarction and is helpful in identifying the responsible artery. Diffusion-weighted imaging (DWI) sequence can indicate the position of the acute infarct, while cases of splenic infarction can be easily diagnosed via CTA. For children with limb arterial embolism, ultrasonography is the recommended examination, given it can detect the formation of lower limb arteriovenous thromboses. If necessary, low tube voltage paired with low contrast CTA can provide a more comprehensive view of limb vessel arteries.

The first-line treatment for pediatric patients with acute PE is anticoagulant therapy. The American Society of Hematology (ASH) guideline panel recommends using anticoagulation therapy over no anticoagulation therapy in pediatric patients with symptomatic deep vein thrombosis (DVT) or PE. Both anticoagulation- and no anticoagulation-based therapies are recommended for use in pediatric patients with asymptomatic DVT or PE. The ASH guideline panel advises against using thrombolysis followed by anticoagulation; rather, anticoagulation alone should be used in pediatric patients with DVT. The recommendation panel also discourages the use of thrombolysis followed by anticoagulation; rather, anticoagulation alone should be used in pediatric patients with submassive PE. It recommends thrombolysis followed by anticoagulation, rather than anticoagulation alone, in pediatric patients with PE and hemodynamic compromise. Furthermore, the ASH guideline panel does not recommend using thrombectomy followed by anticoagulation; rather, anticoagulation alone should be used in pediatric patients with symptomatic DVT or PE. Besides, the inferior vena cava (IVC) filter should not be used; anticoagulation alone should be used in pediatric patients with symptomatic DVT or PE. Anticoagulation should be used for ≤ 3 months rather than > 3 months in pediatric patients with provoked DVT or PE; anticoagulation should be used for 6 to 12 months rather than for > 6 to 12 months in pediatric patients with unprovoked DVT or PE [43].

There is no consensus regarding the treatment of MP pneumonia complicated with intracardiac thrombosis. Treatment options mainly include anticoagulant therapy, thrombolytic therapy, and surgical thrombectomy. Thrombolytic therapy of intracardiac thrombus in children is in its infancy and warrants further research. Herein, only 2 of the 14 children with cardiac thrombosis were treated with urokinase thrombolysis (initial 4400 IU/kg administered in 10 min, then 4400 IU/kg/h, q12h, for 5 days). The remaining children were treated with low molecular weight heparin calcium anticoagulation (200 IU/kg/d, q12h) and oral aspirin (4 mg/kg/day) to inhibit platelet aggregation. The general course of treatment for heparin drugs is 5–10 days. Attention should be paid to monitoring APTT during this time. Moreover, vitamin K antagonists should be used after 12–48 h of heparin treatment. The most extensively used antagonist is oral warfarin for ≤ 3 months. The international standardized ratio should be monitored during this period and generally maintained at 2–3 [43, 44]. Most patients with acute pulmonary embolism receive appropriate anticoagulant therapy and have a favorable prognosis, but caution is warranted about the thrombus disintegrates and causes a new pulmonary embolism during treatment. One case of pulmonary embolism complicated with right ventricular thrombosis developed shoulder pain, and irritability, among other symptoms during treatment. Cardiac ultrasound revealed that the thrombus adhering to the cardiac wall fell off. The symptoms of the child improved after about 1 h and was free from dyspnea, chest pain, hemoptysis, and other manifestations, and the prognosis was satisfactory. For pulmonary embolism and cardiac thrombosis, the time of thrombus disappearance was followed up for 1–3 months. Data on the prognosis of pulmonary embolism are scarce. A study conducted in Canada on the follow-up of 405 children with pulmonary embolism and deep venous thrombosis evinced that the child mortality rate was 16% [45]. Except for 3 cases of external cerebral embolism with apparent sequelae, the remaining 11 cases had a good curative effect, and there were no mortalities.

RMPP with a high level of D-dimer and inflammatory markers were strongly associated with thrombosis. Symptoms associated with thrombosis, such as chest pain, may be subtle, and patients may be asymptomatic. Therefore, keeping a high index of suspicion for thrombosis in children with RMPP is critical. Contrast-enhanced lung CT, echocardiography, and blood vessel ultrasonography should be routinely performed in RMPP patients with a high level of D-dimer and inflammatory markers.

## Data Availability

All data generated or analysed during this study are included in this published article.
